# Characterizing the detection of inactivated *Mycoplasma hyopneumoniae* DNA in the respiratory tract of pigs

**DOI:** 10.1186/s13567-024-01273-2

**Published:** 2024-02-15

**Authors:** Albert Canturri, Maria Pieters

**Affiliations:** 1 Department of Veterinary Population Medicine, College of Veterinary Medicine, University of Minnesota, St. Paul, MN USA; 2 Veterinary Diagnostic Laboratory, College of Veterinary Medicine, University of Minnesota, St. Paul, MN USA; 3 Swine Disease Eradication Center, College of Veterinary Medicine, University of Minnesota, St. Paul, MN USA

**Keywords:** *Mycoplasma hyopneumoniae*, PCR, diagnostics, pigs, DNA, bacterial DNA

## Abstract

A positive *Mycoplasma hyopneumoniae* PCR result in a clinical specimen may eventually represent the mere detection of non-viable bacteria, complicating the diagnostic interpretation. Thus, the objective of this study was to evaluate the PCR detection of non-viable *M. hyopneumoniae* and its residual cell-free DNA in live pigs. Pigs were inoculated with either active or inactivated *M. hyopneumoniae* and were sampled for up to 14 days. *Mycoplasma hyopneumoniae* was not detected by PCR at any timepoint in pigs inoculated with the inactivated bacterium, suggesting that in healthy pigs, the non-viable *M. hyopneumoniae* DNA was rapidly sensed and cleared.

## Introduction, methods, and results

*Mycoplasma hyopneumoniae* (*M. hyopneumoniae*), a respiratory bacterial pathogen that attaches to and disturbs ciliated epithelial cells of the airways, causes a highly prevalent disease in pigs named enzootic pneumonia [1]. Due to its intrinsic fastidiousness to grow in culture, PCR is the assay of choice to detect the presence of this pathogen in a specimen and thus, a positive PCR result would be interpreted as that the pig from which the sample was collected was infected with *M. hyopneumoniae*. However, one of the most important disadvantages of this diagnostic approach is that PCR can detect nucleic acids from non-viable bacteria [2], potentially misleading the interpretation.

Animal taxonomy and evolutionary biology have been revolutionized by the analysis and characterization of DNA fragments from archeological bones tens of thousands and even millions of years old [3]. Thus, there is no doubt that DNA is a robust and persistent molecule ex-vivo, even when subjected to long periods of unfavorable environmental conditions.

The persistence of nucleic acid detection from non-viable cells in vivo, although less studied, has been also reported. For instance, microbial cell-free DNA (cfDNA), a highly fragmented nucleic acid released from decomposing cells, can be identified by PCR in specific organs and in the blood [4]. Specifically, cfDNA from respiratory pathogens has been detected in cystic fibrosis (CF) human patients [5]. In CF, only a subset of the bacterial communities trapped in the mucus are viable and metabolically active and thus, DNA-based detection methods such as PCR are not suitable to track changes in microbiota composition and metabolism [6, 7]. Similarly, it has been shown that bacterial DNA on the skin surface overrepresents the viable skin microbiome [8]. In veterinary medicine, the detection of RNA from a bluetongue virus inactivated vaccine product in the blood of sheep up to nine days after injection has been described using real-time RT-PCR [9], as well as in the blood and spleen of cattle [10]. These findings emphasize the diagnostic challenge that the PCR detection of non-viable pathogens can pose, especially in field applications, such as in the evaluation of antibiotic treatment efficacy or in the determination of bacterial clearance post-infection.

It is currently unknown if, and for how long, the DNA from non-viable *M. hyopneumoniae* can be detectable by PCR in vivo, in the respiratory airways of pigs. Therefore, the objective of this study was to assess the PCR detection dynamics of non-viable *M. hyopneumoniae* DNA in experimentally inoculated pigs.

This study was conducted according to a protocol approved by the Institutional Animal Care and Use Committee of the University of Minnesota. Sixteen pigs from a *M. hyopneumoniae* and Porcine Reproductive and Respiratory Syndrome Virus (PRRSV) negative farrow-to-finish farm were randomly selected, at four weeks of age, and transported to the veterinary isolation facilities at the University of Minnesota. Negative status of the source farm was based on historical diagnostic results regarding *M. hyopneumoniae* seroconversion and detection by PCR, as well as lack of disease-associated clinical signs. Pigs were confirmed negative to *M. hyopneumoniae* via species-specific real-time PCR [11] and ELISA testing (Idexx, Westbrook, Maine, USA) prior to experimental inoculation.

A lung homogenate containing 1 × 10^5.5^ color changing units/mL of *M. hyopneumoniae* strain 232 (purchased from Iowa State University, Ames, IA, USA) and Friis medium  was used as the inoculum for this study, which was administered in either an active or an inactivated form. The active inoculum, containing viable bacteria, was maintained at 80°C and prepared just minutes prior to inoculation. The inactivated inoculum, containing non-viable *M. hyopneumoniae,* was treated via autoclaving at 121 °C and 15 psi for 40 min, and stored at −20 °C until use. Both, the active and inactivated inocula were tested by PCR [11] prior to use and showed a Ct value of 28.47 and 29.00, respectively*. Mycoplasma hyopneumoniae*-specific bacterial culture was performed on the inactivated inoculum to determine the efficacy of the heat and pressure treatment on viability. Culture conditions consisted of incubation of original and diluted material in liquid medium (ML, Mycoplasma Experience LTD, Surrey, UK) at 37 °C in agitation (100 rpm) for at least seven days. Growth of *M. hyopneumoniae* was not obtained from the autoclaved inoculum.

**Figure 1:**
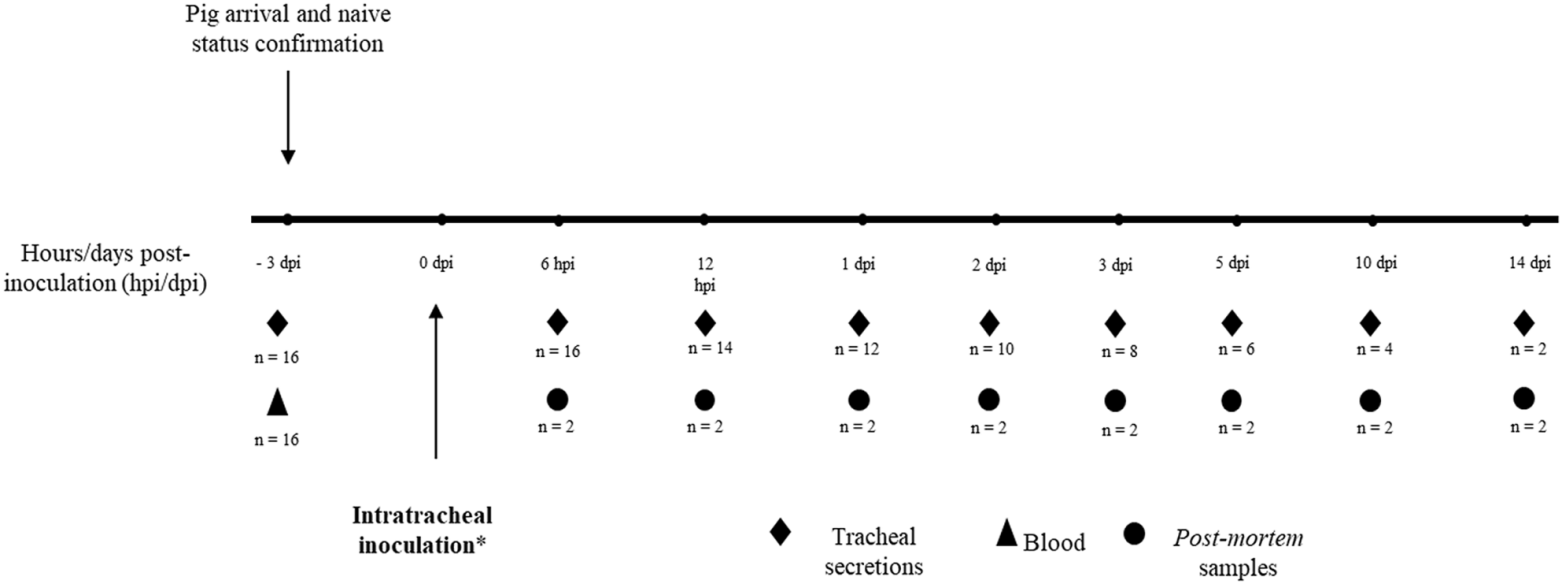
**Experimental design and sample collection scheme**. *Eight pigs were inoculated with inactivated *Mycoplasma hyopneumoniae*, while eight pigs were inoculated with the viable bacterium. Two pigs were humanely euthanized at every time point (one pig per experimental group). Black diamonds indicate ante-mortem tracheal secretions sample collection. Black triangle indicates blood sample collection. Black circles indicate post-mortem sample collection at necropsy, including tracheal scrapings, bronchial secretions, bronchoalveolar lavage fluid, thoracic lymph nodes and spleen. Macroscopic and microscopic lung lesion assessment and grading was performed in all euthanized pigs.

The experimental design of this study is graphically depicted in Figure 1. All pigs in the study were three-week old females. At time 0, eight pigs were intratracheally inoculated [12] with 10 mL of the inactivated *M. hyopneumoniae* and transferred to a different experimental room. Likewise, eight pigs were inoculated with a similar volume of the active *M. hyopneumoniae* and were transferred to a different experimental room. At 6 and 12 h post-inoculation (hpi), and at 1, 2, 3, 5, 10, and 14 days post-inoculation (dpi), tracheal secretions (ante-mortem samples) were obtained from each live pig, as described by Fablet et al. [13]. A different set of sterile materials and surgical gloves were used for each individual pig at each sampling event. Researchers collecting samples showered in, and changed clothes and PPE prior to entering each of the two different experimental rooms. Additionally, one pig per group was humanely euthanized at each timepoint and a necropsy was performed. Samples obtained *post-mortem* included tracheal mucosa (obtained via cell scraping), broncho-alveolar lavage fluid (BALF), bronchial secretions (obtained via swabbing), tracheobronchial and mediastinal lymph nodes and spleen. All samples intended to perform PCR testing were frozen at −20 °C until processing. A portion of the right apical lung lobe of each pig was collected and fixed in 10% buffered formalin. Lungs were macroscopically assessed at necropsy and lesions morphologically compatible with pulmonary consolidation were scored based on the percentage of lobe area affected, as described by Straw et al. [14]. Histopathologic analysis was also performed, and lesions indicative of mycoplasmal pneumonia were scored from 0 to 4, per the scoring system described by Woolley et al. [15]. Both macroscopic and microscopic lesion scorings were performed by the same board-certified pathologist while pig identification was masked. Nucleic acids were extracted by using the MagMAX™ CORE Nucleic Acid Purification Kit coupled with MagMAX^™^ Express-96 Magnetic Particle Processor (Life Technologies, Grand Island, NY, USA). Real-time PCR was performed using the VetMAX™-Plus qPCR Master Mix and VetMAX™ *M. hyopneumoniae* Reagents Kit (Life Technologies, Grand Island, NY, USA), according to the manufacturer’s instructions.

**Table 1:** **Macroscopic percentage of lung with pulmonary consolidation and histopathologic severity of** ***Mycoplasma hyopneumoniae*-characteristic lesions.**

		Time post-inoculation							
		6 h	12 h	1 d	2 d	3 d	5 d	10 d	14 d
Inactivated inoculum	Macroscopic score (%)	0	0	0	1	0	0	0	0
	Histopathologic score (0–4)	0	0	0	0	0	0	0	0
Active inoculum	Macroscopic score (%)	0	0	0	0	0	0	32	16
	Histopathologic score (0–4)	0	0	0	0	0	0	2	3

Lung lesion scores for pigs euthanized at different times post-inoculation. One pig from each experimental group, inoculated with either the inactivated or the active inoculum, was euthanized at each time point and a post-mortem lung evaluation was performed. h:  hours. d:  days.

**Figure 2:**
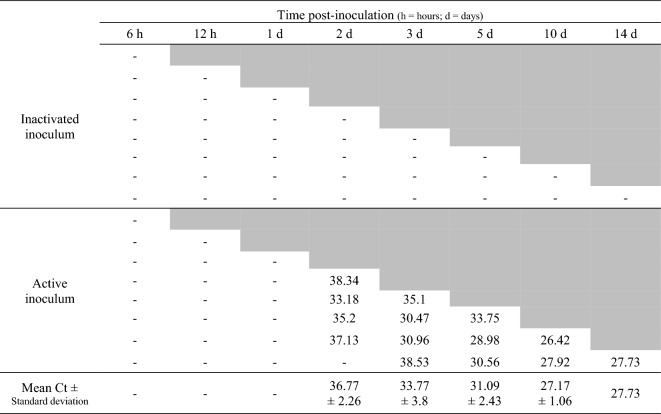
Ante-mortem detection of *Mycoplasma hyopneumoniae* by PCR in tracheal secretion samples. Each row represents an individual pig. A dash represents target not detected, while numbers represent Ct values. Gray shaded cells represent samples not collected (pigs euthanized at a previous timepoint).

**Figure 3:**
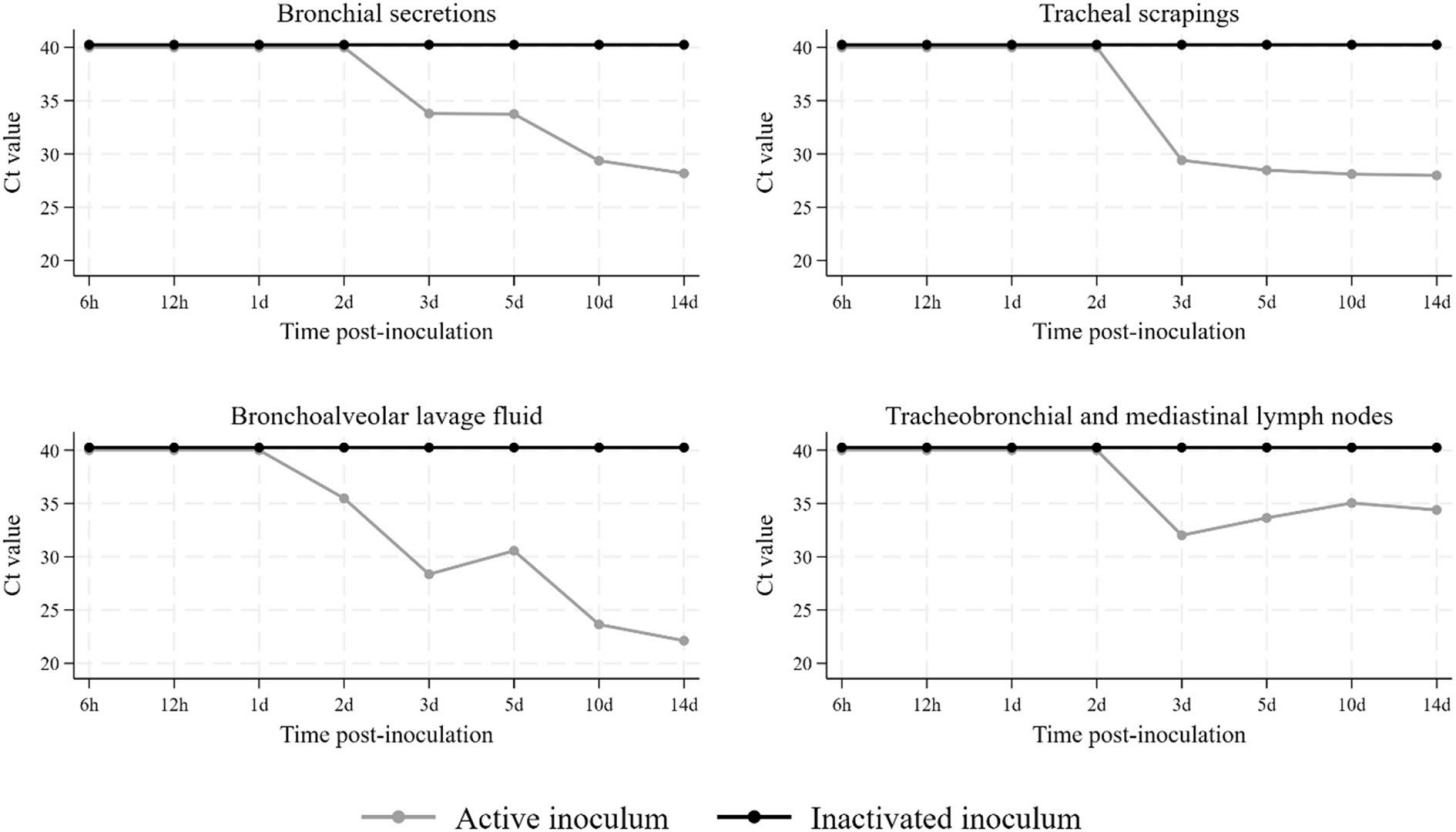
Post-mortem detection of *Mycoplasma hyopneumoniae *by PCR in various sample types. Results are expressed as Ct values. Gray dots: Active inoculum. Black dots: Inactivated inoculum.

Detection of *M. hyopneumoniae* DNA by PCR was not obtained at any time point in pigs inoculated with inactivated bacteria, either in samples collected *ante-mortem* (Figure 2) or post-mortem (Figure 3). In pigs inoculated with the active bacterium, *M. hyopneumoniae* was detected by PCR starting at 2 dpi in tracheal secretions and BALF, and at 3 dpi in bronchial secretions, tracheal scrapings and thoracic lymph nodes. PCR detection in pigs inoculated with viable *M. hyopneumoniae* was observed until the final collection time point, at 14 dpi (Figures 2, 3). *Mycoplasma hyopneumoniae* PCR detection was not obtained from the spleen of any of the pigs in the study. Neither macroscopic nor histologic lung lesions compatible with *M. hyopneumoniae* infection were observed in pigs receiving the inactivated inoculum. Cranioventral pulmonary consolidation and *M. hyopneumoniae*-characteristic lesions were macroscopically and histologically observed at 10 and 14 dpi in pigs inoculated with the active bacterium (Table 1). Pigs inoculated with either inactivated or viable *M. hyopneumoniae* exhibited histologic lesions consistent with diffuse lymphohistiocytic interstitial pneumonia with arteritis/periarteritis from 6 hpi to 3 dpi.

## Discussion

This study evaluated the PCR detection dynamics of non-viable *M. hyopneumoniae* cells and their residual nucleic acids in the respiratory system of live pigs. Based on the fact that there is no direct relationship between the viability of bacteria and their detection by PCR, it is conceptually possible that a positive PCR result in a clinical specimen represents the mere detection of residual DNA from non-viable microorganisms. In the case of *M. hyopneumoniae*, since culture-dependent methods to assess bacterial viability are impractical, PCR has become the diagnostic assay of choice. Nevertheless, result interpretation can be challenging in certain epidemiologic scenarios. For example, in pigs at chronic stages of disease that have overcome clinical presentation, the detection of *M. hyopneumoniae* by PCR could indicate the presence of viable bacteria that have not yet been cleared from the respiratory tract and may potentially be infectious to others. Alternatively, *M. hyopneumoniae* detection by PCR may represent the presence of DNA remnants of non-viable bacteria that have not yet been cleared. Thus, positive pigs would no longer be truly colonized with viable *M. hyopneumoniae* and would not represent a risk of infection to other pigs.

Results obtained in this study indicate that, in pigs with non-compromised mucociliary apparatuses and immune systems, the DNA of non-viable *M. hyopneumoniae* was rapidly sensed and cleared, being not detectable by PCR in the respiratory or the lymphatic systems at all time points post intratracheal inoculation. On the contrary, in pigs inoculated with viable *M. hyopneumoniae*, DNA was consistently detected by PCR in different parts of the respiratory system starting at days two or three post-inoculation.

Rapid recognition of an invading pathogen by a host is the first step to create a protective immune response. Different components of the innate immune system cooperate to avoid or minimize the establishment of infectious agents and the development of their detrimental effects. The mucociliary system is one of the first defense mechanisms for invading respiratory pathogens, which become trapped within the mucus layer and are expelled out of the airways by the synchronized movement of cilia. The function of the mucociliary system can be compromised when pigs are exposed to respiratory infections, most notably *M. hyopneumoniae*, toxic gases such as ammonia, or dust [16]. Pigs in the present study harbored intact mucociliary clearance apparatuses, potentially minimizing the contact between *M. hyopneumoniae* DNA and the mucosa of the airways and propelling it out of the respiratory system. Other key components of the innate immune response are the different families of pattern recognition receptors (PRRs), such as toll-like receptors (TLRs), that are capable of recognizing foreign DNA, particularly TLR9 [17, 18]. TLR2 and TLR6 have been also described to be involved in the recognition of *M. hyopneumoniae* by porcine alveolar macrophages [19]. Thus, the presence of DNA and cellular debris from degenerated *M. hyopneumoniae* was probably detected by resident cells of the respiratory innate immune system, such as dendritic cells and macrophages, and rapidly cleared. Additionally, both the active and inactivated inocula used in this experiment contained Friis medium, which is supplemented, among other components, with yeast extract and both equine and swine serum, substances that are most likely immunogenic when introduced to the respiratory tract of pigs, exacerbating the response of the immune system. A likely immunogenic response was evidenced by the presence of lymphohistiocytic interstitial pneumonia with arteritis/periarteritis in the lungs of pigs exposed to either type of inoculum (i.e. active or inactivated) within the first three days. Altogether, these mechanisms most likely contributed to non-detectability of the DNA of non-viable *M. hyopneumoniae*. However, pigs housed in commercial farms are commonly exposed to environmental conditions and bacterial infections that weaken the mucociliary apparatus, as well as viral pathogens, such as PRRSV or porcine circovirus, which are known to have an immunomodulatory role, delaying host immune responses [20]. Thus, the persistence of genetic material from non-viable *M. hyopneumoniae* in pigs housed in farm conditions and subject to coinfections with other pathogens may be extended and hence, further research is warranted.

Interestingly, there was lack of detection of the bacterium by PCR in the first two days post-inoculation, even in samples from the lower respiratory tract and lymphoid system collected post-mortem in pigs exposed to the live bacterium. Similar results were previously reported in nasal and laryngeal secretions and tracheo-bronchial lavage fluid at 2 dpi, and became positive at 5 dpi [21]. The cause of the transient lack of detectability of live *M. hyopneumoniae* early post-inoculation, as well as the location of *M. hyopneumoniae* within the respiratory system during that time is unknown, and the significance of this finding is, at this moment, uncertain. It is important to note that this was a pilot study, and thus, sample size was limited, which could have an effect on the reproducibility of the obtained data.

In summary, results from this investigation suggest that only viable *M. hyopneumoniae* could be detected by PCR in the respiratory tract of otherwise healthy pigs two days post-inoculation. Ideally, a culture-independent molecular diagnostic method, such as one detecting exclusively viable *M. hyopneumoniae* can aid investigate the eventual persistence of non-viable bacterial cells in the respiratory system. Such viability assay could be applied in situations where treatments or management practices are expected to affect the viability of *M. hyopneumoniae* in vivo.

## Data Availability

The datasets used and/or analyzed during the current study are available from the corresponding author on reasonable request.
